# Effectiveness and Safety of Image-Guided Renal Biopsies: Insights from 5,235 Procedures in the German Society for Interventional Radiology and Minimally Invasive Therapy (DeGIR) Registry

**DOI:** 10.1007/s00270-025-04313-2

**Published:** 2025-12-29

**Authors:** R. Ocker-Serger, M. Opitz, L. Klüner, D. Rosok, M. Drews, Y. Thal, M. Forsting, J. Haubold, J. Nadjiri, B. M. Schaarschmidt, S. Zensen

**Affiliations:** 1 Institute of Diagnostic and Interventional Radiology and Neuroradiology, University Hospital Essen, Essen, Germany; 2 Department of Interventional Radiology, Klinikum Rechts Der Isar, Technical University of Munich, Munich, Germany

**Keywords:** Biopsy, Needle, Kidney/pathology, Image-guided biopsy, Treatment outcome, Postoperative complications

## Abstract

**Purpose:**

This study evaluates the technical success, diagnostic yield, and complication rates of image-guided percutaneous renal biopsies based on multicenter registry data from the German Society for Interventional Radiology and Minimally Invasive Therapy.

**Materials and Methods:**

This retrospective analysis included 5,235 renal biopsies at 176 centers in Germany, Austria, and Switzerland between 2018 and 2024. Technical success was defined as image-guided confirmed needle placement within the target lesion. Diagnostic yield is defined as proportion of procedures providing adequate samples for clinical diagnosis.

**Results:**

CT was used for image guidance in 78.53% followed by ultrasound in 12.32%. Technical success was 98.38% and diagnostic yield 94.92%. Technical success was high in both inpatients (98.33%) and outpatients (99.26%; OR = 0.44, 95%CI 0.11–1,80; *p* = 0.241). The overall complication rate was 5.04%, with major complications in 0.74%. Complications were more frequent with pathological platelet counts and INR values, but none of these coagulation parameters remained independently associated in multivariable analysis. CT-guided biopsies showed higher complication rates than ultrasound-guided procedures, and this association remained after adjustment (adjusted OR 8.57, 95%CI 3.40–21.58; *p* < 0.001)**.** No fatal complications occurred within 24 h; however, three delayed deaths were documented among hospitalized patients.

**Conclusion:**

Percutaneous image-guided renal biopsies are effective and safe, with low complication rates and high diagnostic yield. Ultrasound guidance remained independently associated with a lower complication risk, likely reflecting selection bias.

**Graphical abstract:**

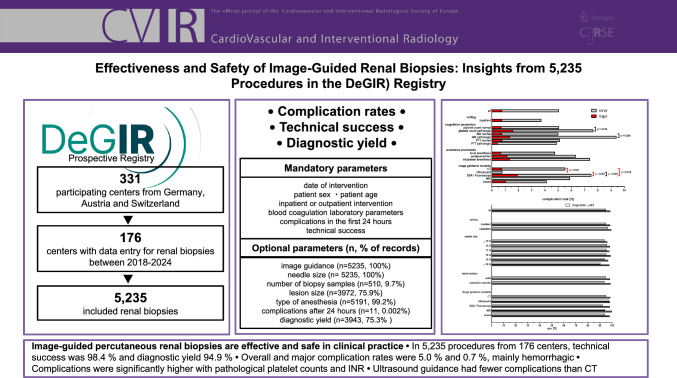

## Introduction

Percutaneous image-guided renal biopsy plays a central role in the diagnostic workup of renal parenchymal diseases and indeterminate renal masses. With the growing use of targeted therapies and immune checkpoint inhibitors in nephrology and oncology, obtaining adequate tissue for histological classification and molecular profiling has become increasingly important. Imaging modalities, while indispensable for lesion detection and localization, are often insufficient to reliably distinguish between inflammatory, infectious, and neoplastic processes—particularly when treatment decisions hinge on precise histopathological subtypes or molecular alterations [1, 2].

Histopathological evaluation therefore remains the diagnostic gold standard in many renal conditions and is central to precision medicine approaches [3].

Renal biopsies are typically performed under ultrasound (US) or computed tomography (CT) guidance, with the choice of modality depending on lesion visibility, anatomical location, and operator expertise. According to current Guidelines, percutaneous renal mass biopsy is recommended in selected clinical situations, including active surveillance of small renal masses, preablation evaluation, or histological confirmation of metastases [4], and may directly impact treatment [5].

Despite increasing clinical relevance, large-scale multicenter data on safety and diagnostic performance remain limited with published evidence derived from single-center series or prospective cohorts with predominantly US-guided procedures [6–8].

The German Society for Interventional Radiology and Minimally Invasive Therapy (DeGIR), a member of the Cardiovascular and Interventional Radiological Society of Europe (CIRSE), maintains a prospective multicenter registry for quality assurance and research, currently encompassing 331 centers across Germany, Austria, and Switzerland. This provides a robust platform for evaluating clinical routine in interventional radiology [9–12].

The aim of this study was to analyze the technical success, diagnostic yield, and complication rates of image-guided percutaneous renal biopsies across DeGIR registry sites between 2018 and 2024.

## Material and Methods

### Data Source and Study Design

This retrospective multicenter study included image-guided percutaneous renal biopsies documented in the prospectively DeGIR registry between January 2018 and December 2024. Ethical approval for the registry was obtained centrally, with additional local approval. Participating centers entered standardized routine data via a Web-based platform (samedi GmbH, Berlin, Germany).

### Inclusion Criteria and Definitions

**Fig. 1:**
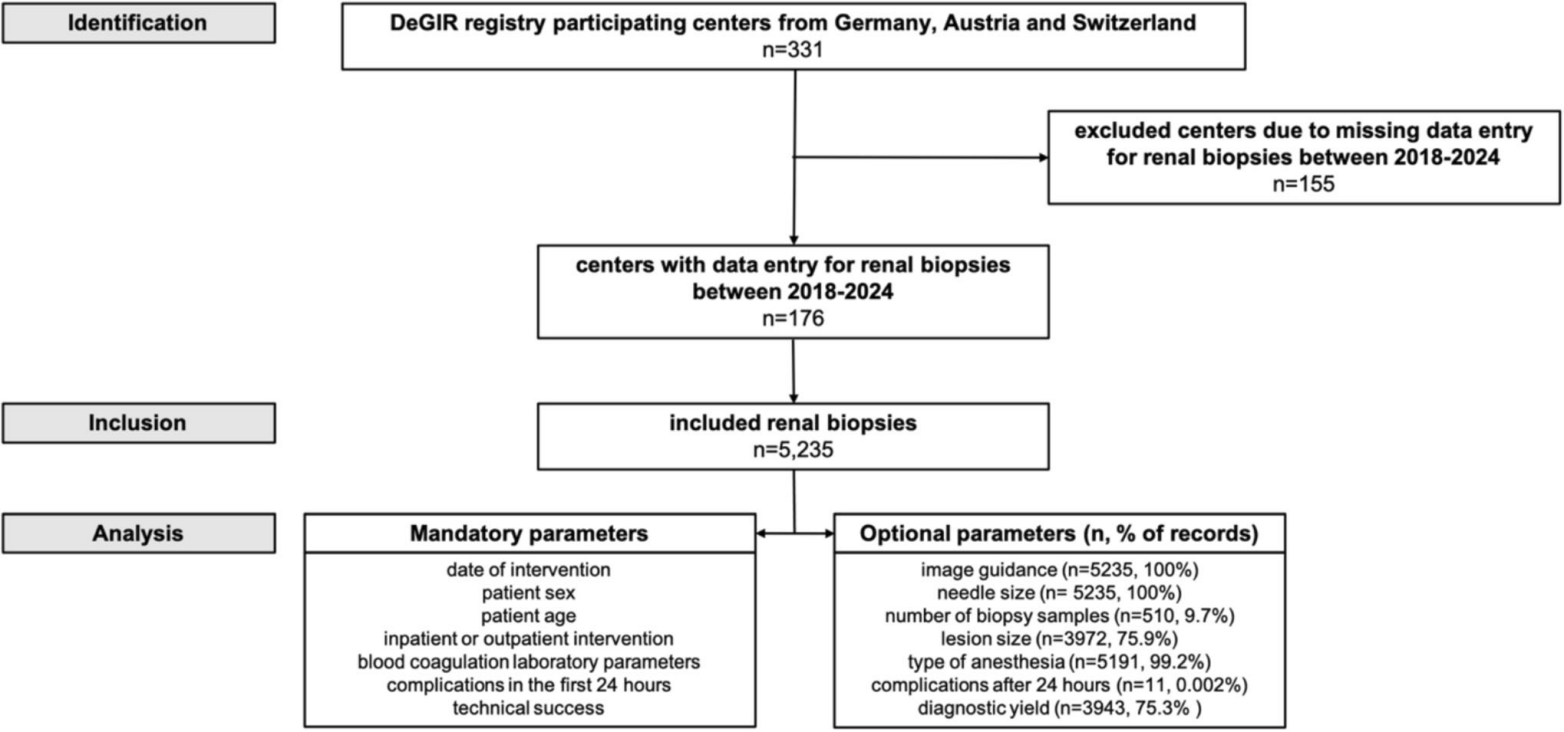
Study flowchart

The dataset comprised both mandatory and optional variables, as outlined in Fig. 1. Technical success was defined as visually confirmed biopsy needle placement within the target lesion. The coagulation parameters (platelet count, INR, PTT) were recorded as either pathological or normal, based on the local laboratory standards of the individual participating centers. No predefined numerical thresholds were available in the registry.

Complications were classified according to the Society of Interventional Radiology (SIR) severity grading system, distinguishing between minor (grades A–B) and major (grades C–F) events [13], and categorized as either early (within 24 h) or delayed (onset after 24 h). The updated CIRSE classification system [14] represents the current standard for complication grading in interventional radiology.

Diagnostic yield was defined as the proportion of procedures that resulted in a histologically adequate sample enabling a definitive clinical diagnosis. As histological reports were documented only when entered as an optional registry field, diagnostic yield was calculated from the subset of cases with available reports (*n* = 4,154). Lesion texture was classified at the time of biopsy according to imaging appearance. Cases with missing data were excluded from the respective analyses.

### Statistical Analysis

Statistical analysis was performed using SPSS software, version 26.0 (IBM Corp., Armonk, NY, USA). The distribution of continuous variables was assessed with the Kolmogorov–Smirnov test. Non-normally distributed variables are reported as median with interquartile range (IQR). Categorical variables, including technical success, diagnostic yield, and complication rates, were compared using the Chi-square test. Non-normally distributed continuous variables between groups were compared using the Mann–Whitney U test. Binary univariable and multivariable logistic regression models were performed to identify independent predictors of complications. A *p*-value < 0.05 was considered statistically significant. All subgroup analyses were exploratory, and no formal adjustment for multiple testing was applied. Reported p-values should therefore be interpreted descriptively.

## Results

### Patient Characteristics

**Fig. 2:**
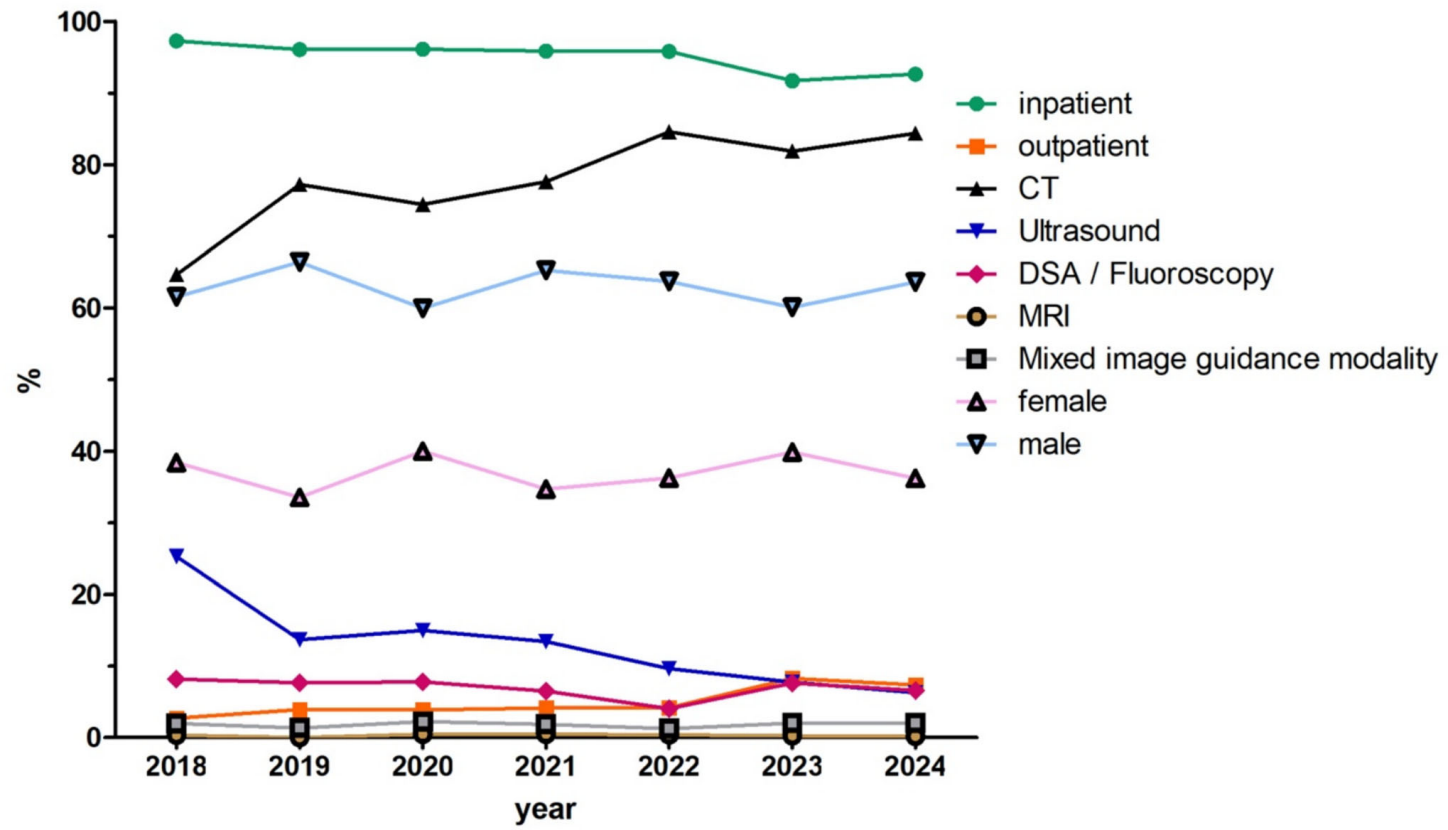
Frequency of percutaneous image-guided renal biopsies from 2018 to 2024 by setting, gender distribution, and image guidance modality

Between 2018 and 2024, a total of 5,235 image-guided percutaneous renal biopsies were reported to the DeGIR registry by 176 centers (Fig. 1). The median number of procedures per center was 8 (IQR: 3–32). The majority of procedures were performed in male patients. The median patient age was 70 years (IQR: 60–80). Information on interdisciplinary tumor board decisions was available for 7.49% of procedures (392/5,235), and in all documented cases, the biopsy had been recommended by the board. Most procedures were performed in inpatients (94.9%), with an increasing proportion of outpatient biopsies (Table 1, Fig. 2).

**Table 1:** Frequency of percutaneous image-guided renal biopsies from 2018 to 2024 by setting, gender distribution, and image guidance modality

	2018	2019	2020	2021	2022	2023	2024	All
**All**	549	664	794	795	714	784	935	5235
	10.49%	12.68%	15.17%	15.19%	13.64%	14.98%	17.86%	100%
**Setting**								
Inpatient	534	638	763	762	684	719	866	4966
	97.27%	96.08%	96.10%	95.85%	95.80%	91.71%	92.62%	94.86%
Outpatient	15	26	31	33	30	65	69	269
	2.73%	3.92%	3.90%	4.15%	4.20%	8.29%	7.38%	5.14%
**Gender distribution**								
Female	211	223	318	276	259	313	339	1939
	38.43%	33.58%	40.05%	34.72%	36.27%	39.92%	36.26%	37.04%
Male	338	441	476	519	455	471	595	3295
	61.57%	66.42%	59.95%	65.28%	63.73%	60.08%	63.64%	62.94%
**Image guidance modality**								
CT	355	513	591	617	604	642	789	4111
	64.66%	77.26%	74.43%	77.61%	84.59%	81.89%	84.39%	78.53%
US	139	91	119	107	69	61	59	645
	25.32%	13.70%	14.99%	13.46%	9.66%	7.78%	6.31%	12.32%
DSA/fluoroscopy	45	51	62	52	29	60	62	361
	8.20%	7.68%	7.81%	6.54%	4.06%	7.65%	6.63%	6.90%
MRI	2	0	4	4	3	2	2	17
	0.36%	0%	0.50%	0.50%	0.42%	0.26%	0.21%	0.32%
Mixed	11	9	18	15	9	16	19	97
	2.00%	1.36%	2.27%	1.89%	1.26%	2.04%	2.03%	1.85%

### Lesion and Biopsy Characteristics

CT was the predominant image guidance modality (78.53%), followed by US, DSA/fluoroscopy, MRI, and mixed guidance (Table 1). The median lesion size was 26 mm (IQR: 16–43 mm). For CT-guided procedures, the median lesion size was 29 mm (IQR: 20–45), for US-guided procedures 30 mm (IQR: 20–55 mm; p = 0.139). Most lesions were solid (75.30%, 3,942/5,235). The number of biopsy samples was documented in 9.74% (510/5,235) of all procedures, with a median number of 3 samples (IQR: 2–3). Among the documented cases, in 35.67% (182/510), only a single biopsy sample was obtained. In 81.96% (418/510), up to 3 biopsy samples were obtained. 1.45% (76/5,235) of procedures were discontinued, most commonly due to patient noncooperation (50.0%, 38/76), miscellaneous reasons (23.68%, 18/76), or technical/ anatomical difficulties (18.42%, 14/76). Intervention-dependent complications led to discontinuation in only 1.32% (1/76). Nearly all discontinued procedures (97.37%) occurred in an inpatient setting (74/76) (Table 2).

**Table 2:** Complications within 24 h of percutaneous image-guided renal biopsies

	**Procedures (n)**	**Complications total (n [%])**	**Major according to SIR classification **[13]** (n [%])**
All	5235	264	39
		5.04%	0.74%
**Setting**			
Outpatient	269	10	2
		3.72%	0.74%
Inpatient	4966	254	37
		5.11%	0.75%
**Lesion Size**			
< 10 mm	117	6	1
		5.13%	0.85%
10–19 mm	796	49	10
		6.16%	1.26%
20–29 mm	1043	49	7
		4.70%	0.67%
30–39 mm	698	33	0
		4.73%	0%
40–49 mm	432	31	2
		7.18%	0.46%
50–59 mm	285	17	5
		5.96%	1.75%
> 60 mm	602	25	4
		4.15%	0.66%
Not specified	1262	54	10
		4.28%	0.79%
**Needle Size**			
14 G	173	6	6
		3.47%	3.47%
15 G	49	4	0
		8.16%	0%
16 G	635	30	4
		4.72%	0.63%
17 G	380	27	2
		7.11%	0.53%
18 G	3747	186	29
		4.96%	0.77%
19 G	90	5	2
		5.56%	2.22%
20 G	120	6	2
		5%	1.67%
21 G	41	0	0
		0%	0%
**Lesion texture**			
Solid	3942	188	29
		4.77%	0.74%
Subsolid	143	12	1
		8.39%	0.69%
Necrotic	304	19	1
		6.25%	0.33%
Mixed texture	93	8	1
		8.6%	0.01%
Unspecified	753	37	7
		4.91%	0.93%
**Coagulation-related laboratory parameters**			
Platelet count normal	4577	231	32
		5.05%	0.70%
Platelet count pathological	380	29	6
		7.63%	1.58%
International normalized ratio (INR) normal	4811	240	36
		4.99%	0.75%
International normalized ratio (INR) pathological	224	21	3
		9.38%	1.34%
Partial thromboplastin time (PTT) normal	4732	242	37
		5.11%	0.78%
Partial thromboplastin time (PTT) pathological	246	12	1
		4.88%	0.41%
**Anesthesia procedures**			
Local anesthesia	4694	223	32
		4.75%	0.68%
Analgosedation	348	22	4
		6.32%	1.15%
Intubation anesthesia	149	11	2
		7.38%	1.34%
Unspecified	44	1	1
		2.27%	2.27%
**Image guidance modality**			
CT	4111	227	31
		5.52%	0.75%
Ultrasound	645	5	0
		0.78%	0%
DSA/fluoroscopy	361	27	7
		7.48%	1.94%
MRI	17	1	0
		5.88%	0%
Mixed	97	4	1
		4.12%	1.03%

### Complications

**Fig. 3:**
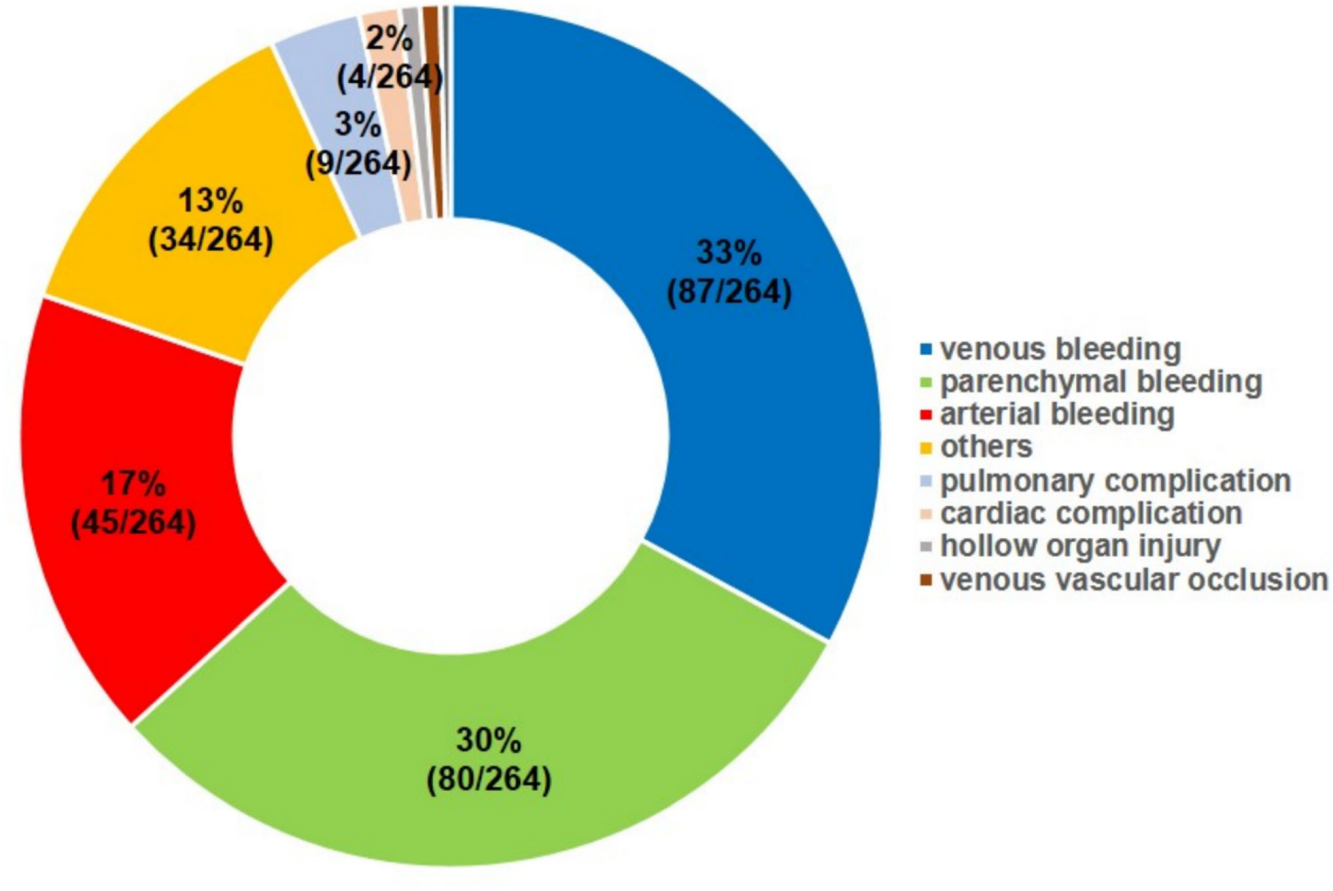
Complications of percutaneous image-guided renal biopsies differentiated by complication type

The overall complication rate within 24 h post-procedure was 5.04%. Most complications were minor (4.30%), including 59.11% (133/225) classified as SIR grade A (no therapy, no sequelae) and 40.89% (92/225) as grade B (requiring symptomatic therapy or observation). Major complications occurred in 0.74%, including 69.23% grade C (27/39) and 30.77% grade D complications (12/39). There was no permanent health damage (Grade E) or death (Grade F) within 24 h. The most frequent complication type was bleeding (Fig. 3). The cause of bleeding (venous, parenchymal, arterial) was documented in the register by the attending physician; the diagnostic method was not specified.

A total of 0.21% (11/5.235) delayed complications (> 24 h post-procedure) were reported, of which 36.36% were classified as minor (4/11) and 63.64% as major (7/11). All delayed complications were observed in the inpatient setting, with 27.3% (3/11) resulting in death (Grade F).

When comparing image guidance modalities, CT-guided biopsies were associated with significantly higher overall complication rates than US-guided procedures (5.52% vs. 0.78%, *p* < 0.001). Major complications occurred significantly more frequently with CT guidance than with US guidance (0.75% vs. 0%, OR = 0.99, 95%CI 0.99–0.995; *p* = 0.027). In the multivariable logistic regression including measurable confounders, CT guidance remained the only independent predictor of complications compared to US (adjusted OR 8.57, 95%CI 3.40–21.58; *p* < 0.001, Table 3). When comparing fluoroscopy with CT- and US-guided procedures, fluoroscopy was associated with significantly higher major complication rates (CT vs. fluoroscopy major: OR = 0.38, 95%CI 0.17–0.88; p = 0.019; US vs. fluoroscopy, p < 0.001 for both overall (OR = 0.1, 95%CI 0.04–0.25) and major complications (OR = 1.02, 95%CI 1.01–1.04). Fluoroscopy did not remain a significant predictor after adjustment for confounders.

**Table 3:** Baseline predictors of complications within 24 h following percutaneous image-guided renal biopsies: results from univariate and multivariable logistic regression

Predictor	Category (reference)	Complications, n (%)	Univariable OR	95% CI	*p*-value	Multivariable OR	95% CI	*p*-value
**Setting**	Inpatient (ref.) Outpatient	254 (5.11%) 10 (3.72%)	1.396	0.733–2.659	0.310	1.420	0.740–2.726	0.292
**Lesion Size**								
	< 10 mm	6 (5.13%)	1.285	0.169–9.774	0.809	0.702	0.394–1.250	0.229
	10–19 mm	49 (6.16%)	0.694	0.433–1.112	0.129	1.281	0.167–9.834	0.811
	20–29 mm	49 (4.70%)	0.944	0.589–1.514	0.812	0.738	0.456–1.195	0.217
	30–39 mm	33 (4.73%)	0.921	0.552–1.534	0.751	0.980	0.607–1.582	0.934
	40–49 mm	31 (7.18%)	0.589	0.350–0.993	**0.047**	0.946	0.564–1.585	0.832
	50–59 mm	17 (5.96%)	0.756	0.417–1.369	0.356	0.627	0.369–1.063	0.083
	> 60 mm (ref.)	25 (4.15%)						
	not specified	54 (4.28%)	1.027	0.648–1.630	0.909	0.781	0.428–1.424	0.420
**Needle Size**								
	14 G	6 (3.47%)	1.332	0.485–3.656	0.578	3.209	0.777–13.263	0.107
	15 G	4 (8.16%)	0.588	0.209–1.651	0.313	1.305	0.469–3.635	0.610
	16 G	30 (4.72%)	1.053	0.710–1.564	0.797	0.744	0.252–2.194	0.592
	17 G	27 (7.11%)	0.683	0.450–1.037	0.074	1.023	0.682–1.534	0.913
	18 G (ref.)	186 (4.96%)						
	19 G	5 (5.56%)	0.888	0.356–2.215	0.799	0.763	0.497–1.170	0.215
	20 G	6 (5.00%)	0.992	0.431–2.285	0.986	1.122	0.430–2.932	0.814
**Lesion texture**								
	Solid (ref.)	188 (4.77%)						
	Subsolid	12 (8.39%)	0.547	0.297–1.005	0.052	0.983	0.590–1.640	0.948
	Necrotic	19 (6.25%)	0.751	0.462–1.222	0.250	0.535	0.281–1.020	0.057
	Mixed texture	8 (8.60%)	0.538	0.357–1.127	0.101	0.789	0.479–1.301	0.354
	Unspecified	37 (4.91%)	0.968	0.674–1.389	0.859	0.671	0.311–1.445	0.308
**Coagulation-related laboratory parameters**								
	Platelet count normal (ref.)	231 (5.05%)						
	Platelet count pathological	29 (7.63%)	0.641	0.429–0.958	**0.030**	3.374	1.008–11.298	0.049
	International Normalized Ratio (INR) normal (ref.)	240 (4.99%)						
	International Normalized Ratio (INR) pathological	21 (9.38%)	0.508	0.318–0.810	**0.004**	3.540	0.749–16.731	0.111
	Partial thromboplastin time (PTT) normal (ref.)	242 (5.11%)						
	Partial thromboplastin time (PTT) pathological	12 (4.88%)	1.051	0.580–1.904	0.870	0.392	0.165–0.931	0.034
**Anesthesia procedures**								
	Local anesthesia (ref.)	223 (4.75%)						
	Analgosedation	22 (6.32%)	0.763	0.486–1.199	0.242	2.420	0.323–18.110	0.390
	Intubation anesthesia	11 (7.38%)	0.646	0.345–1.211	0.173	0.662	0.413–1.062	0.087
	Unspecified	1 (2.27%)	2.216	0.304–16.160	0.433	0.707	0.374–1.336	0.286
**Image guidance modality**								
	CT (ref.)	227 (5.52%)						
	Ultrasound	5 (0.78%)	7.461	3.064–18.171	** < 0.001**	8.566	3.400–21.580	** < 0.001**
	MRI	1 (5.88%)	0.877	0.115–6.670	0.899	0.921	0.120–7.069	0.937
	DSA/fluoroscopy	27 (7.48%)	0.721	0.477–1.091	0.122	0.823	0.516–1.312	0.413
	Mixed	4 (4.12%)	1.506	0.550–4.125	0.426	1.763	0.619–5.020	0.289

**Fig. 4:**
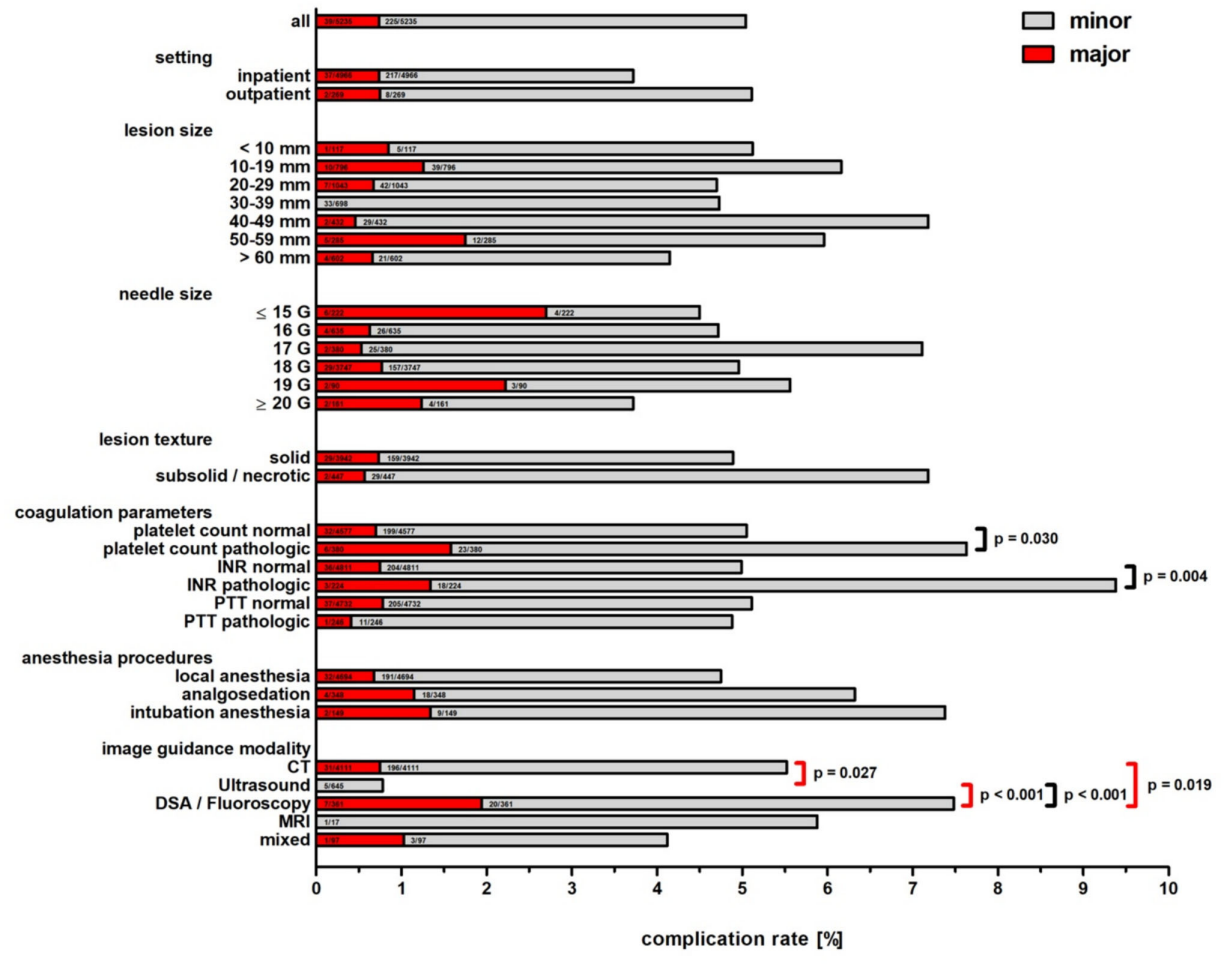
Complications of percutaneous image-guided renal biopsies, including significant p-values

The overall complication rates did not differ significantly between inpatient and outpatient procedures (OR = 0.72, 95%CI 0.38–1.36; *p* = 0.308). Major complication rates were identical in both settings (OR = 1.00, 95%CI 0.24–4.18; *p* = 0.998, Fig. 2). Lesion size did not significantly influence complication risk. Pathological platelet counts (7.63% vs. 5.05%; OR = 1.55, 95%CI 1.04–2.32; *p* = 0.030) and pathological INR values (9.38% vs. 4.99%; OR = 1.97, 95%CI 1.23–3.15; *p* = 0.004) were associated with higher complication rates, but not in those with pathological PTT (4.88% vs. 5.11%; OR = 0.95, 95%CI 0.53–1.72; *p* = 0.870). After adjustment, none of these coagulation parameters remained independently associated with complications (Table 3). Rates of major complications showed no significant differences across these groups (Table 2, Fig. 4).

Compared to procedures performed under local anesthesia, procedures under analgosedation and general anesthesia showed numerically higher complication rates; however, none of these differences reached statistical significance (all *p* > 0.14).

### Technical Success

**Fig. 5:**
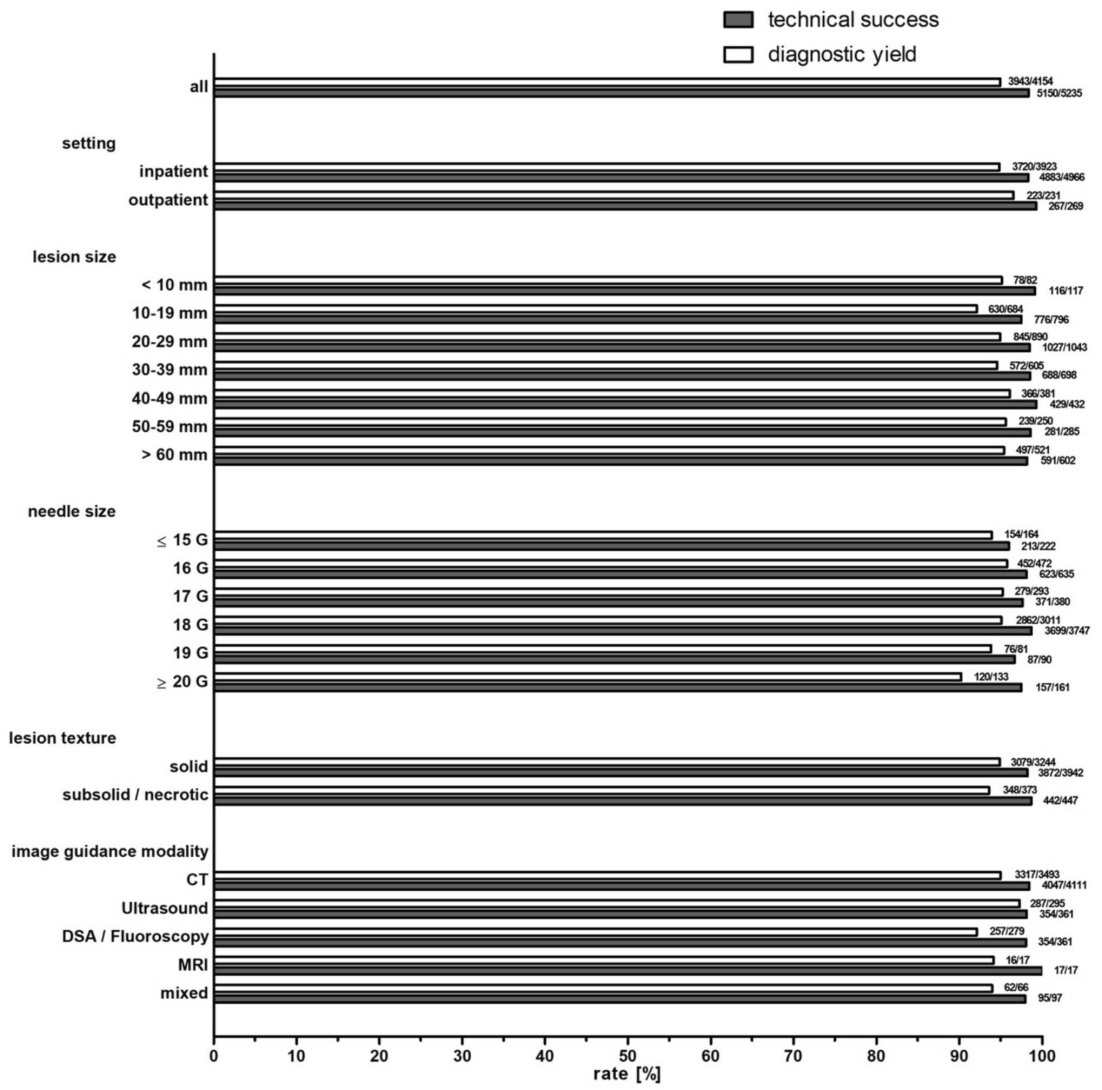
Technical success and histological representativeness rates of percutaneous image-guided renal biopsies

The overall technical success rate was 98.38%. Technical success rates were equally high for CT guidance and for US guidance (OR = 1.2, 95%CI 0.64–2.23; *p* = 0.567), for inpatients and outpatients (OR = 0.44, 95%CI 0.11–1.80; *p* = 0.241) as well as solid and subsolid lesion texture (OR = 0.39, 95%CI 0.05–2.82; *p* = 0.333). No consistent association was found between needle size and technical success (Table 4, Fig. 5).

**Table 4:** Technical success and diagnostic yield of percutaneous image-guided renal biopsies

	**Procedures**	**Technical success**		**Diagnostic yield**		
	n	Procedures (n)	Rate (%)	Report available (n)	Diagnosis possible (n)	Rate (%)
**All**	5235	5150	98.38	4154	3943	94.92
**Setting**						
Outpatient	269	267	99.26	231	223	96.54
Inpatient	4966	4883	98.33	3923	3720	94.83
**Lesion size**						
≤ 10 mm	117	116	99.15	82	78	95.12
10–19 mm	796	776	97.49	684	630	92.11
20–29 mm	1043	1027	98.47	890	845	94.94
30–39 mm	698	688	98.57	605	572	94.55
40–49 mm	432	429	99.31	381	366	96.06
50–59 mm	285	281	98.60	250	239	95.60
≥ 60 mm	602	591	98.17	521	497	95.39
not specified	1262	1242	98.42	741	716	96.63
**Needle Size**						
≤ 14 G	173	165	95.38	129	122	94.57
15 G	49	48	97.96	35	32	91.43
16 G	635	623	98.11	472	452	95.76
17 G	380	371	97.63	293	279	95.22
18 G	3747	3699	98.72	3011	2862	95.05
19 G	90	87	96.67	81	76	93.83
20 G	120	119	99.17	98	90	91.84
≥ 21 G	41	38	92.68	35	30	85.71
**Lesion texture**						
Solid	3942	3872	98.22	3244	3079	94.91
Subsolid	143	142	99.30	115	107	93.04
Necrotic	304	300	98.68	258	241	93.41
Mixed	93	91	97.85	83	79	95.18
Unspecified	753	744	98.80	453	436	96.25
**Number of biopsies**						
1	182	182	100	123	115	93.50
2	110	110	100	74	71	95.95
3	126	126	100	99	98	98.99
4	44	44	100	31	31	100
5	19	19	100	16	16	100
6	22	22	100	15	15	100
≥ 7	7	7	100	5	4	80
Not specified	4725	4640	98.20	3791	3593	94.78
**Anesthesia procedures**						
Local anesthesia	4694	4617	98.36	3734	3539	94.78
Analgosedation	348	342	98.28	256	251	98.05
Intubation anesthesia	149	149	100	123	117	95.12
Unspecified	44	42	95.45	41	36	87.80
**Image guidance modality**						
CT	4111	4047	98.44	3493	3317	94.96
Ultrasound	645	633	98.14	295	287	97.29
DSA/fluoroscopy	361	354	98.06	279	257	92.11
MRI	17	17	100	17	16	94.12
Mixed	97	95	97.94	66	62	93.94

### Diagnostic Yield

Diagnostic yield was high for CT and US guidance (OR = 0.53, 95%CI 0.26–1.08; *p* = 0.074). The overall diagnostic yield was 94.92%, whereby outpatients achieve similar rates to inpatients (OR = 1.52, 95%CI 0.74–3.12; *p* = 0.250).

Subsolid and necrotic had slightly, not significantly lower diagnostic yield compared to solid lesions (subsolid: OR = 0.72, 95%CI 0.34–1.49; *p* = 0.373; necrotic: OR = 0.76, 95%CI 0.45–1.27; *p* = 0.295). Diagnostic yield was highest in lesions measuring 40–49 mm (96.06%, 366/381) and lowest in lesions between 10 and 19 mm (92.11%, 630/694, Table 4). No consistent association was found between needle size and diagnostic yield (Table 4, Fig. 5).

The number of biopsy samples was reported in 9.74% of procedures (510/5,235). Among these, diagnostic yield increased with the number of biopsy samples obtained, reaching 99% (98/99) with three samples and 100% with four to six samples (62/62).

## Discussion

This large-scale registry analysis provides important insights into the current use, effectiveness, and safety of image-guided renal biopsies: Reported biopsies showed consistently high technical success and diagnostic yield rates, underscoring their established diagnostic role in nephrologic and oncologic care. The overall complication rate was low (5.04%), with major complications occurring in only 0.74% of cases, confirming a favorable safety profile. The proportion of outpatient procedures—although still limited—steadily increased over the study period, reflecting growing confidence in decentralized biopsy strategies and supporting their feasibility in selected patients.

Bleeding accounted for the majority of complications, reflecting the kidney’s dense vascularization and limited compressibility in the retroperitoneum. Importantly, major complications were rare (0.74%), and no fatal events occurred within 24 h, in line with recent meta-analyses and national registries [6, 7, 15, 16].

US guidance was independently associated with a lower complication risk compared with CT, even after adjustment for measurable confounders. Although this finding supports guideline recommendations favoring US guidance whenever feasible, causal inference is limited by the observational design and possible preferential use of US in technically less complex cases; residual selection bias cannot be excluded. Fluoroscopy-guided renal biopsy showed significantly higher major complication rates in univariable analysis, consistent with current guidelines that no longer recommend fluoroscopy as a standard modality [17].

Patients with coagulation abnormalities, particularly pathological platelet count or INR, had higher complication rates in univariable analyses. The findings are consistent with previous institutional reports [6, 16] and reinforce consensus guidelines that emphasize the importance of thorough preprocedural coagulation screening and correction [17, 18]. The SIR guideline further recommends individualized risk assessment and defines specific thresholds for platelet count and INR and provides detailed protocols for managing anticoagulation in image-guided interventions [19, 20]. Even after adjustment, none of these coagulation parameters remained independently associated with complications, and our findings emphasize that adherence to these standards is essential for procedural safety.

Lesion size, including lesions < 10 mm, did not significantly affect complication rates. These data align with previous studies suggesting that small lesion size does not inherently increase procedural risk, provided appropriate technique and imaging guidance are used [21–23]. Thus, lesion size alone should not preclude biopsy when histological diagnosis is clinically indicated.

Technical success was equally high for both CT- and US-guided biopsies, indicating that both modalities are reliable when lesions are adequately visualized. Technical success rates were consistently high in both outpatient and inpatient procedures, which supports the feasibility and safety of renal biopsy in selected outpatient procedures. Previous studies and guideline recommendations indicate that the vast majority of patients are suitable for outpatient renal biopsy. Hospitalization should be restricted to rare cases with unmodifiable anticoagulation or severe coagulation disorders [24–26].

Subsolid or necrotic lesions had slightly lower diagnostic yield compared to solid lesions, likely due to sampling of non-viable, hemorrhagic, or acellular tissue—an effect that has been reported in prior studies [21, 27].

A very high diagnostic yield (62/62 cases) was observed when 4–6 cores were obtained. This association should be interpreted with caution, given the relatively small subgroup size and the observational nature of our study; causality cannot be inferred. This observation supports the EAU guideline recommendation to retrieve at least two good quality biopsy samples, and avoid necrotic areas to maximize diagnostic yield [4]. In nephrologic indications, recommendations suggest retrieving enough tissue to evaluate approximately 10–20 glomeruli for sample adequacy in renal biopsies [8, 28]. This approach supports both diagnostic reliability and procedural safety. While excessive sampling could theoretically increase bleeding risk, adequate tissue sampling should be prioritized, especially in oncologic or complex nephrologic cases.

As a registry-based study, our analysis is subject to certain limitations. Data completeness depended on site-reported entries, and optional parameters were not uniformly documented. Selection bias cannot be fully excluded, especially for outpatient procedures, which may have been performed in lower-risk patients. Furthermore, the number of outpatient biopsies was relatively low (n = 269 compared to 4,966 inpatient procedures), which led to limited statistical significance for subgroup comparisons. All delayed events (> 24 h) occurred in hospitalized patients, which is likely due to differences in monitoring rather than an actual absence of events in the outpatient group; this detection bias must be taken into account when interpreting the safety data. No central histopathological review was available and complications were classified according to the SIR rather than the newer CIRSE-based standards. Procedural details such as post-biopsy observation time, specific biopsy indication, and operator-level factors were not captured, and thresholds for defining pathological coagulation parameters may have varied between centers. Underreporting cannot be excluded, representing a potential reporting bias. Nonetheless, the large sample size, multicenter scope, and real-world nature of the dataset offer valuable insights into routine practice across a broad healthcare landscape.

## Conclusion

Image-guided percutaneous renal biopsy is a safe and highly effective diagnostic tool across a wide spectrum of clinical settings. The low rate of major complications—even in routine clinical settings—highlights its favorable safety profile, particularly when coagulation parameters are within recommended limits. With appropriate patient selection, outpatient biopsy is feasible, without compromising safety or diagnostic quality. Adequate tissue sampling and strict adherence to coagulation management protocols remain critical to ensure diagnostic utility and minimize procedural risk. These real-world data contribute to refining procedural standards and support the broader implementation of renal biopsy in the era of precision medicine.
